# CD133 promotes gallbladder carcinoma cell migration through activating Akt phosphorylation

**DOI:** 10.18632/oncotarget.7474

**Published:** 2016-02-18

**Authors:** Chen Li, Cong Wang, Yang Xing, Jiaojiao Zhen, Zhilong Ai

**Affiliations:** ^1^ Zhongshan Hospital of Fudan University, Shanghai, People's Republic of China; ^2^ Key Laboratory of Glycoconjuates Research, Ministry of Public Health, Department of Biochemistry and Molecular Biology, Shanghai Medical College of Fudan University, Shanghai, People's Republic of China

**Keywords:** CD133, gallbladder carcinoma, migration, invasion, Akt pathway

## Abstract

Gallbladder carcinoma (GBC) is the fifth most common malignancy of gastrointestinal tract. The prognosis of gallbladder carcinoma is extremely terrible partially due to metastasis. However, the mechanisms underlying gallbladder carcinoma metastasis remain largely unknown. CD133 is a widely used cancer stem cell marker including in gallbladder carcinoma. Here, we found that CD133 was highly expressed in gallbladder carcinoma as compared to normal tissues. CD133 was located in the invasive areas in gallbladder carcinoma. Down-regulation expression of CD133 inhibited migration and invasion of gallbladder carcinoma cell without obviously reducing cell proliferation. Mechanism analysis revealed that down-regulation expression of CD133 inhibited Akt phosphorylation and increased PTEN protein level. The inhibitory effect of CD133 down-regulation on gallbladder carcinoma cell migration could be rescued by Akt activation. Consistent with this, addition of Akt inhibitor Wortmannin markedly inhibited the migration ability of CD133-overexpressing cells. Thus, down-regulation of CD133 inhibits migration of gallbladder carcinoma cells through reducing Akt phosphorylation. These findings explore the fundamental biological aspect of CD133 in gallbladder carcinoma progression, providing insights into gallbladder carcinoma cell migration.

## INTRODUCTION

Gallbladder carcinoma is an aggressive malignancy and carries an extremely poor prognosis [1, 2]. This poor survival rate is because of the early spread of tumors via lymphatic, perineural, and hematogenous routes [3, 4]. Thus, exploring novel signal molecules involved in gallbladder carcinoma metastasis may provide new effective therapeutic strategies.

There is now compelling evidence that the bulk of the malignant cells in cancers are generated by cancer stem cells [5]. Furthermore, cancer stem cell is supposed to be responsible for cancer metastasis. CD133/Prominin-1, a five transmembrane glycoprotein, is first reported to be found in epithelium, and then in hematopoietic system [6–8]. Recent years, CD133 has been widely used as a cell surface marker to identify and to isolate cancer stem cells from various cancer tissues [9–11]. Increasing evidence has shown that CD133 contributes to cancer growth and invasion. For example, knockdown of CD133 decreases the growth of human hepatocellular carcinoma cells [12–14]. CD133 promotes colon cancer cell proliferation [15]. Down-regulation of CD133 expression inhibits the self-renewal and tumorigenesis of glioma stem cells [16]. These findings raise the possibility that CD133 is a molecular target for effective cancer therapies.

CD133 has also been used as a marker to isolate cancer stem cell in gallbladder carcinoma. CD133+ gallbladder carcinoma cells possessed high colony-formation ability and higher tumorigenicity than the CD133- population [11]. Our previously also showed purified CD133+ gallbladder carcinoma cells are highly resistant to conventional chemotherapy [17]. However, the contribution of CD133 in gallbladder carcinoma remains largely unknown. Here, we showed that CD133 was highly expressed in gallbladder carcinoma as compared to normal tissues. Down-regulation expression of CD133 inhibited migration and invasion of gallbladder carcinoma through reducing Akt phosphorylation. These finding explores the fundamental biological aspect of CD133, providing insights into gallbladder carcinoma cell migration.

## RESULTS

### CD133 is highly expressed in gallbladder carcinoma and located in the invasive area in gallbladder carcinoma

**Figure 1:**
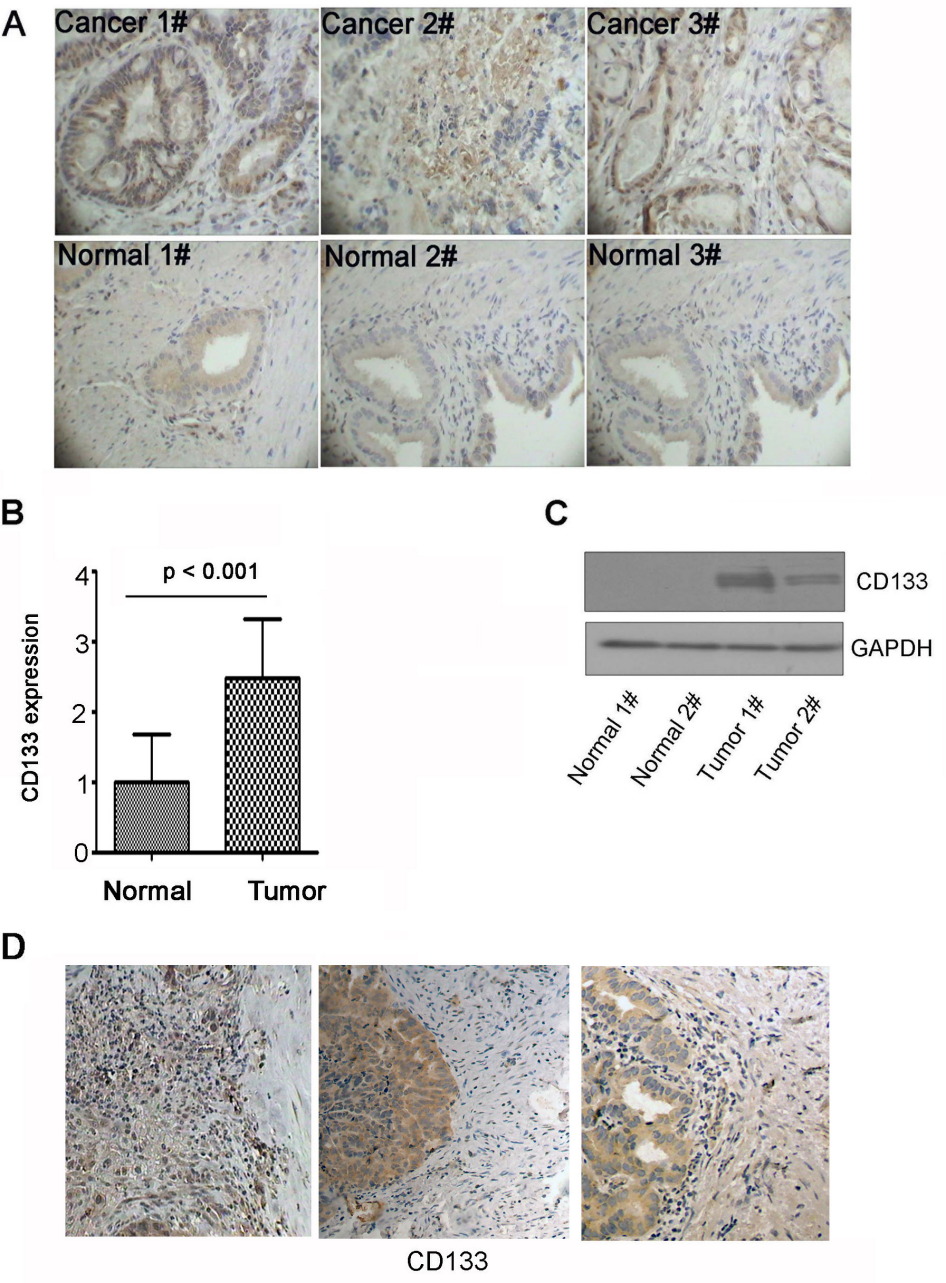
CD133 is highly expressed in gallbladder carcinoma and locates in the invasive areas in gallbladder carcinoma (**A–B**) Immunohistochemistry for CD133 expression in gallbladder carcinoma (Cancer) and normal gallbladder tissue (Normal). Representative pictures (Magnification 200 ×) (A) and quantification of CD133 expression (B). (**C**) Western blots analysis of CD133 expression in gallbladder carcinoma and normal gallbladder tissue. GAPDH served as a loading control. (**D**) Representative immunohistochemical staining of CD133 in gallbladder carcinoma which had clearly identifiable borders between tumor mass and normal tissues. Note the location of CD133 in the invasive areas in gallbladder carcinoma.

To examine the role of CD133 in gallbladder carcinoma progression, CD133 protein level was examined in gallbladder carcinoma clinical specimens and normal gallbladder tissues by immunohistochemisty. The mean CD133 expression in gallbladder carcinoma was significantly elevated as compared with NT tissue (*n =* 19, *p* < 0.001) (Figure 1A and 1B; clinical information shown in Table 1). Next, CD133 protein was examined in two cases of NT and gallbladder carcinoma clinical specimens using western blot. CD133 expression in gallbladder carcinoma was significantly elevated as compared with NT tissue (Figure 1C). These data reveal that CD133 is highly expressed in gallbladder carcinoma. Among 19 gallbladder carcinoma specimens, 3 samples had clearly identifiable borders between tumor mass and normal tissues where gallbladder carcinoma invasion had occurred. Strong immunostaining by the anti-CD133 antibody was apparent in the invading gallbladder carcinoma cells (Figure 1D). Thus, strong expression of CD133 in the invasive areas in human gallbladder carcinoma tissue indicates that CD133 is associated with gallbladder carcinoma invasion.

**Table 1:** Relationship between the expression of CD133 protein and histopathologic features of gallbladder carcinoma

Case no.	Age	gender	Tumor stage	Metastasis	CD133 score
1	61	F	I	F	1
2	78	F	I	F	2
3	82	M	IIA	F	1
4	66	M	IIA	F	2
5	75	F	IIIA	F	2
6	45	M	IIIA	F	3
7	66	M	IIIA	F	3
8	64	M	IIIA	F	2
9	46	F	IIIA	F	2
10	66	F	IIIA	F	3
11	62	F	IIIA	F	2
12	73	F	IIIA	F	2
13	46	F	IIIA	F	3
14	69	F	IIIA	F	2
15	77	F	IIIA	F	3
16	79	F	IIIA	T	4
17	58	F	IIIA	T	3
18	59	F	IIIB	T	4
19	78	M	IIIB	T	3

* Immunohistochemical (IHC) staining was estimated as follows: **0**: no signal; **1**: signal i*n* ≤ 10% of cells; **2**: signal in 10%–25% of cells; **3**: signal in 25%–50% of cells; **4**: signal in 50%–75% of cells; **5**: signal in > 75% of cells.

### Down-regulation of CD133 inhibits GBC cell migration

**Figure 2:**
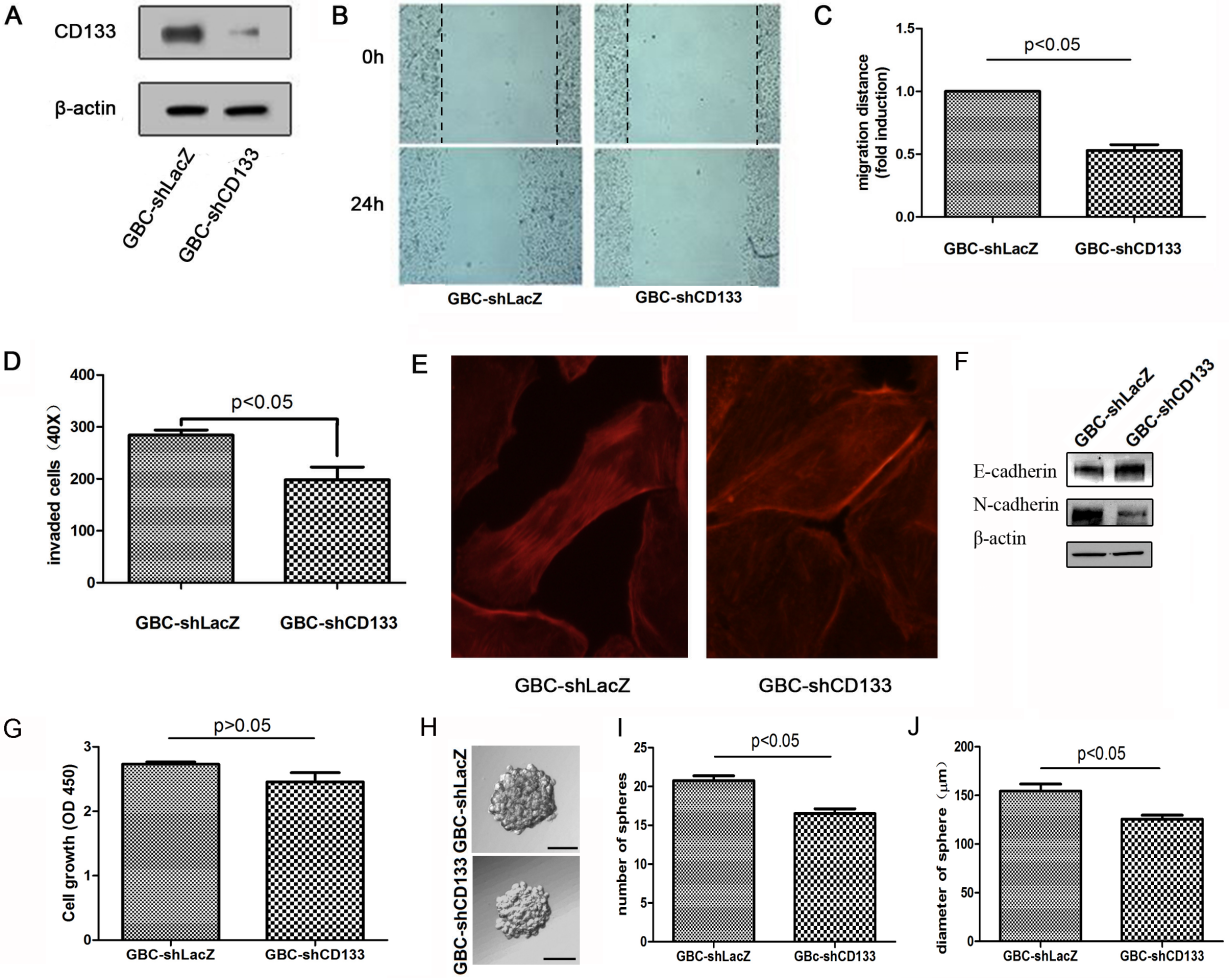
Down-regulation of CD133 suppresses GBC cell migration (**A**) Western blot analysis of CD133 expression in GBC cells infected with control or CD133 shRNA lentivirus. β-actin served as a loading control. (**B–C**) Wound healing migration assay for GBC cells infected with control or CD133 shRNA lentivirus was performed. The healing of wounds by migrated cells at time 0 and 24 was imaged. Representative pictures (B) and quantification of wound-induced migration distance (C). (**D**) Down-regulation of CD133 suppressed GBC cell invason in Transwell assay (*n =* 3). (**E**) GBC cells infected with control or CD133 shRNA lentivirus were subjected to F-actin staining by Rhodamine-phalloidine. (**F**) Western blot analysis of the level of EMT-related markers in GBC cells infected with control or CD133 shRNA lentivirus. β-actin served as a loading control. (**G**) The effect of CD133 knockdown on gallbladder carcinoma cell proliferation as indicated by MTT assay. (**H–J**) Cells were plated in 96-well plates. Representative photographs of spheres formed by cells infected with the corresponding lentivirus (H). The number (I) and diameter (J) of spheres in each well was determined. Values represent means ± SD.

Next, we used a lentiviral shRNA-based system to evaluate the requirement for CD133 in GBC migration. Western blot analysis showed that CD133 expression was obviously reduced by CD133 shRNA lentivirus (Figure 2A). To study the effect of CD133 on GBC cell migration, we performed scratch assays by wounding confluent monolayer of GBC cells infected with LacZ shRNA or CD133 shRNA lentivirus. Wound closure was followed every 24 hours. CD133 down-regulation reduced GBC cell migration (Figure 2B and 2C; *p* < 0.05). Next, we used a Transwell Matrigel invasion assay using conditioned medium as an attractant in the lower chamber. Compared to control cells, down-regulation of CD133 suppressed GBC cell invasion through the Matrigel (Figure 2D). We next determined if differences in actin cytoskeletal organization contributed to the migratory function of CD133. Actin clustered in long filaments along the edges of the control cells but not in the cells infected with CD133 shRNA lentivirus (Figure 2E). Thus, down-regulation of CD133 suppresses GBC cell migration and invasion.

EMT is a critical process playing a critical role in cancer invasion and metastasis [18, 19]. Next, we examined whether CD133 modulated the EMT pathway. Western blot assay displayed that down-regulation of CD133 expression increased an epithelial-like protein expression pattern (E-Cadherin) but decreased a mesenchymal-like protein expression pattern (N-Cadherin) (Figure 2F). We also examined the effect of CD133 down-regulation on cell proliferation. Knockdown CD133 did not obviously decrease gallbladder carcinoma cell proliferation (Figure 2G). Furthermore, we examined the effect of CD133 down-regulation on stemness potential. Knockdown CD133 slightly inhibited the sphere formation ability of gallbladder carcinoma cell (Figure 2H–2J). Together, down-regulation of CD133 mainly suppresses gallbladder carcinoma cell migration.

### Down-regulation of CD133 reduces AKT phosphorylation and increases PTEN protein level

**Figure 3:**
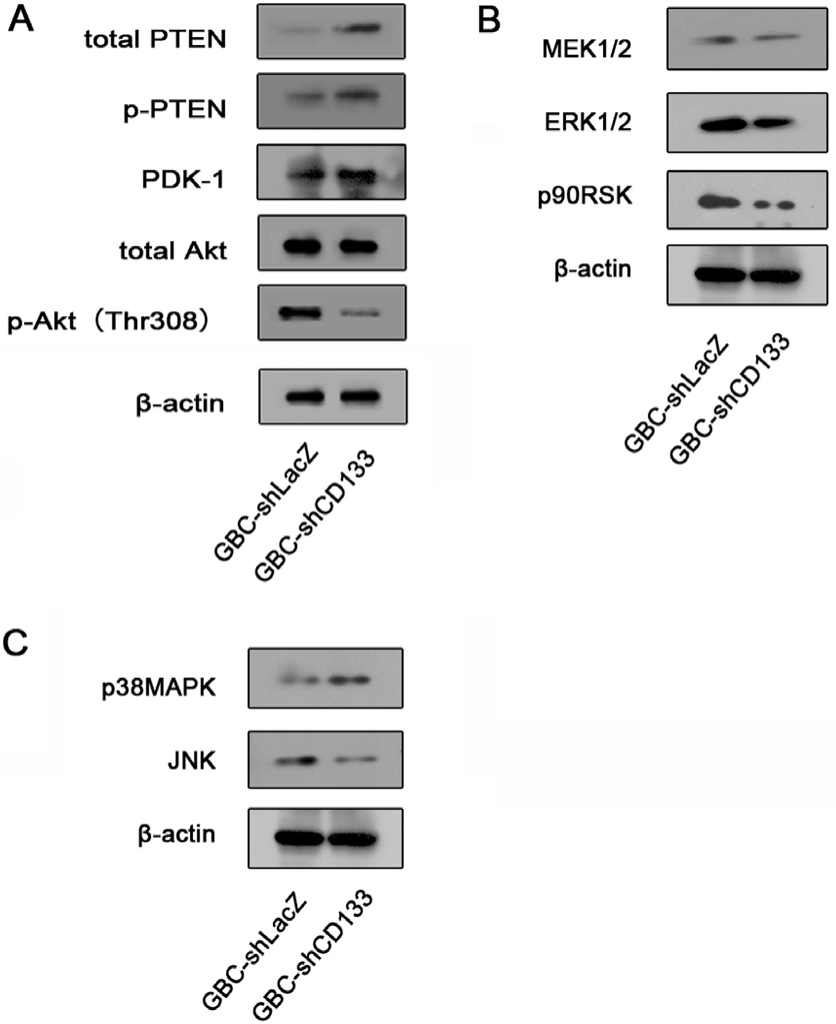
Down-regulation of CD133 reduces Akt phosphorylation (**A**) Western blot analysis of the level of total PTEN, PDK1 and Akt expression and phosphorylation level of Akt and PTEN in GBC cells infected with control or CD133 shRNA lentivirus. β-actin served as a loading control. (**B**) Western blot analysis of the level of total MEK, ERK and p90RSK in GBC cells infected with control or CD133 shRNA lentivirus. β-actin served as a loading control. (**C**) Western blot analysis of the level of total JNK and P38MAPK in GBC cells infected with control or CD133 shRNA lentivirus. β-actin served as a loading control.

We next aimed to explore the mechanisms underlying CD133 promoting cell migration and invasion. We focused on the JNK, ERK and Akt signaling pathways, which have been implicated in regulating cell migration [20–23]. GBC cells infected with LacZ shRNA or CD133 shRNA lentivirus were subjected to western blot analysis for endogenous expression of protein involved in the Akt and ERK signaling cascades. Compared to control cells, GBC expressing CD133 shRNA displayed a marked lower expression of active Akt (phosphorylated at Thr308). Decreased expression of Akt phosphorylation in GBC cells expression CD133 shRNA lentivirus was not a result of a decrease in total Akt protein level (Figure 3A–3C). However, CD133 down-regulation did not significantly reduce the expression of total ERK, JNK or p38MAPK (Figure 3A–3C).

Next, we attempted to explore the mechanism CD133 regulating Akt phosphorylation. PTEN is a phosphatase that dephosphorylates PIP3 to rival the function of PI3K [24]. Increasing evidence indicated that PTEN expression was correlated with CD133 expression [25, 26]. Thus, we examined the effect of CD133 down-regulation on PTEN expression in GBC cells. Down-regulation of CD133 expression increased the expression of PTEN (Figure 3A). Together, down-regulation of CD133 inhibits Akt phosphorylation and increases PTEN expression.

### CD133 promotes gallbladder carcinoma cell migration through activating Akt phosphorylation

**Figure 4:**
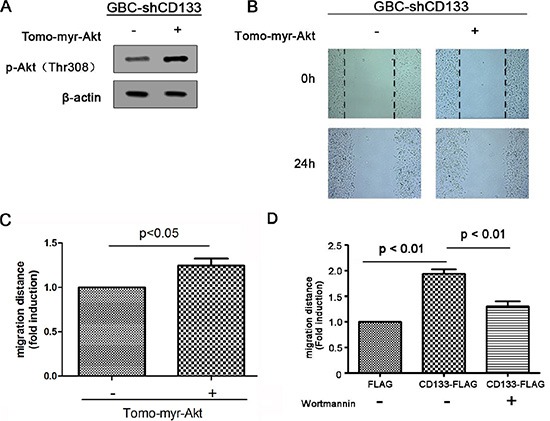
CD133 promotes gallbladder carcinoma cell migration through activating Akt phosphorylation (**A**) Western blots analysis of Akt phosphorylation in CD133 shRNA lentivirus-infected GBC cells transfected with control or Myr-Akt expression vector. β-actin served as a loading control. (**B–C**). Wound healing migration assay for CD133 shRNA lentivirus-infected GBC cells transfected with control or Myr-Akt expression vector. The healing of wounds by migrated cells at time 0 h and 24 h was imaged. Representative pictures (B) and quantification of wound-induced distance (C). (**D**) Wound-healing migration assay for CD133 shRNA lentivirus-infected GBC cells transfected with control or FLAG-CD133 following treated with Akt inhibitor Wortmannin. Quantification of wound-induced migration distance was indicated.

Next, we examined the contribution of Akt signaling pathway in CD133 promoting GBC cell migration. To examine this point, myr-Akt, a constitutively active form of Akt, was introduced to GBC cells infected with CD133 shRNA lentivirus, resulting in up-regulation of Akt phosphorylation (Figure 4A). Myr-Akt inhibited the negative effect of CD133 down-regulation on GBC cell migration (Figure 4B–4C). To further conform that CD133 promotes GBC cell migration depending on activation of Akt phosphorylation, GBC cells expression CD133 shRNA with or without exogenous CD133 were treated by Akt pathway inhibitor Wortmannin. CD133 overexpression obviously promoted GBC cell migration. However, addition of Wortmannin markedly inhibited the migration of CD133-overexpressing cells (Figure 4D). Thus, CD133 promotes gallbladder carcinoma migration at least partly through activation of Akt pathway.

## DISCUSSION

The prognosis of gallbladder carcinoma is terrible partially due to metastasis [3]. Therefore, the identification of novel methods that can effectively inhibit GBC metastasis is needed. Here, we found that down-regulation of CD133 inhibited GBC cell migration and invasion. Consistent with this, ectopic expression of CD133 promoted GBC cell migration. Thus, our data revel that CD133 is a key factor contributing to gallbladder carcinoma migration and invasion. Accumulated evidence from clinical and epidemiological studies has shown that high expression of CD133 is associated with poor prognosis in several different types, including brain tumours, colorectal carcinoma, rectal cancer, hepatoma, gastric carcinoma and medulloblastoma [27–30]. Our data also showed that the expression of CD133 was also significantly increased in gallbladder carcinoma as compared to normal tissues. Thus, CD133 might play functional roles in gallbladder carcinoma progression. However, the mechanism of CD133 up-regulation in gallbladder carcinoma should be further explored.

Another finding was that CD133 promoted gallbladder carcinoma migration at least partly through activation of Akt pathway. The mechanisms by which CD133 promotes tumor cell growth and invasion have been gradually explored. CD133 has been reported to promote cancer cell proliferation through the β-catenin signaling pathway [15]. In glioma, CD133 promotes self-renewal and tumorigenesis of glioma stem cells partly through an interaction between its phosphorylated Y828 residue and the PI3K regulatory subunit p85 [16]. Here, we examined the mechanism by which CD133 promoted gallbladder carcinoma cell migration. Western blot analysis showed that CD133 down-regulation inhibited Akt phosphorylation without changing the level of Akt protein.

Over-activation of Akt is frequently observed in various tumor types and has been reported to be oncogenic [31–33]. Akt pathway modulates cell growth, survival, angiogenesis and mobility [34–37]. Increasing evidence has shown that Akt pathway is uncontrolled in gallbladder carcinoma [17]. Deregulation of PI3K/Akt signaling is sufficient to transform gallbladder epithelial cells [38]. Our finding showed that CD133 was highly expressed in gallbladder carcinoma and activated Akt pathway. These findings might suggest a new mechanism by which Akt pathway is hyper-activated in gallbladder carcinoma.

Another interesting finding was that CD133 knockdown reduced the expression of PTEN. PTEN is a phosphatase that dephosphorylates PIP3 to rival the function of PI3K. Thus PTEN inhibits Akt activation by inhibiting Akt phosphorylation [39]. Wei et. al has reported that CD133 activates PT3K/Akt pathway through an interaction between its phosphorylated Y828 residue and the PI3K regulatory subunit p85 [16]. Our finding might provide a new mechanism by which CD133 activates Akt pathway. The contribution of down-regulation of PTEN in CD133 activating Akt phosphorylation should be further explored. Furthermore, the expression of Akt phosphoarylation or PTEN in gallbladder carcinoma tissues should be examined in future.

In conclusion, we found that CD133 was significantly up-regulated in GBC tissues. Knockdown of CD133 could inhibit GBC cell migration and invasion. Moreover, knockdown of CD133 led to the inactivation of Akt pathway. Therefore, down-regulation of CD133 inhibits migration of gallbladder carcinoma cells at least partly through reducing Akt phosphorylation. These finding might provide a new insight into gallbladder carcinoma migration.

## MATERIALS AND METHODS

### Reagents

Lipofectamine 2000 transfection reagent was obtained from Life Technology. Antibodies to Akt, phosphor-Akt (Thr308), PTEN, phospho-PTEN or β-actin were obtained from Cell Signaling Technology. CD133 antibody was obtained from Miltenyi Biotec. Rhodamine-phalloidine was obtained from Molecular Probes. Matrigel was obtained from Sigma.

### Cell culture

Gallbladder cell line GBC-SD was grown in Dulbecco's modified eagle medium (DMEM high glucose, Gibco, 11965–092) with 10% fetal bovine serum (FBS, Sigma 12003C). Cell line was grown in a humidified incubator supplemented with 5% CO2, at 37°C.

### Scratch assay

For scratch assay, experimental group cell line and control group cell line were cultured in 6-well plate with 10% FBS in DMEM. When 100% confluency had been reached, a linear scarification was made in each well using 1 ml pipet tip across the cell monolayer. Fresh DMEM with 2% FBS was infused. 5 observation points per well were marked and images were taken using an inverted microscope. The cells were then incubated at standard condition for 24 h. Images were taken at the same observation points. Assay was repeated three times.

### Transwell assay

Matrigel (300 ng/ml) was diluted in serum-free DMEM and poured into the upper chambers of a 24-well transwell plate (BD). When Matrigel was solidified, cells were harvested and re-suspended in serum-free DMEM, separately added into the upper chamber with 6 × 10^4^ cells in each chamber. DMEM with 10% FBS was added into the lower chamber of the plate. Cell lines were cultured at standard condition for 48 h. Then, the membranes were fixed in 3.7% paraformaldehyde and stained with crystal violet. Residual in upper chamber was removed by cotton swab. The membranes were washed and dried. Images were taken. Cells on the lower surface were counted. Assay was repeated three times.

### F-actin staining

Cells were fixed in PBS with 3.7% paraformaldehyde for 10 min when confluency reached 80%. PBS was added to rinse cells for three times. 0.1% Triton X-100 in PBS was added and reserved for 5 min. PBS was added to rinse cells for three times. Sealed with 1% BSA in PBS for 30 min. Rhodamine-phalloidine was 200-fold diluted in PBS and 50 μl solution was added onto each cover slip, incubated for 2 h. PBS was added to rinse cells for three times. Images were taken using a laser scanning confocal microscope.

### Western blot

Cells were grown to 75% confluence and were harvested on ice using PBS and lysate was obtained. Lysate was then run out on a SDS-PAGE gel and then transferred onto a 0.4 μm nitrocellulose membrane. Primary antibodies were then added to membrane including anti-CD133 (1:1000), anti-Akt (1:1000), anti-phospho-Akt (Thr308) (1:1000), anti-PTEN (1:1000), anti-phospho-PTEN (1:1000). The proteins were visualized using an ECL system after membrane was then incubated with secondary antibody.

### Immunohistochemical analyzes

This study was approved by the ethics committee of Zhongshan Hospital of Fudan University, and all patients provided informed consent. Tissues used in experiments were fixed in 4% paraformaldehyde, paraffin embedded and sectioned. After deparaffinization, immunohistochemistry was conducted using anti-CD133 antibody antibody (1:100). The images were captured using the Motic Image Advanced 3.2 image analysis system.

### Plasmids construction

To knock down endogenous CD133 expression, the CD133 shRNA lentivirus vectors were generated by ligation of lentivirus vector pLL3.7 with oligonucleotides. The constructs were sequenced, and the correct ones were selected for further experiments.

### Cell proliferation assay

Cells were seeded into 96-well plates in culture medium containing 10% FBS. Cell viability was determined by an MTT assay (wavelength: 450 nm) at various time points.

### Statistic analysis

In general, significance was tested by unpaired two-tailed Student *t* test using GraphPad InStat 5.0 software. The significance of migration and invasion differences in GBC cell lines was determined by using Student *t* test (two-tailed). *P* values < 0.05 were considered statistically significant.
